# A comparison of *in vitro *properties of resting *SOD1 *transgenic microglia reveals evidence of reduced neuroprotective function

**DOI:** 10.1186/1471-2202-12-91

**Published:** 2011-09-23

**Authors:** Siranush A Sargsyan, Daniel J Blackburn, Siân C Barber, Julian Grosskreutz, Kurt J De Vos, Peter N Monk, Pamela J Shaw

**Affiliations:** 1 Department of Medicine, University of Colorado Denver School of Medicine, CO, USA; 2 The Academic Neurology Unit, Sheffield Institute for Translational Neuroscience (SITraN), 385A Glossop Road, University of Sheffield, S10 2HQ, UK; 3 Hans Berger Department of Neurology, University Hospital Jena, Jena, Germany; 4 MRC Centre for Neurodegeneration Research, Institute of Psychiatry, King's College London, London, UK; 5 Department of Infection and Immunity, School of Medicine and Biomedical Sciences, University of Sheffield, UK

## Abstract

**Background:**

Overexpression of mutant copper/zinc superoxide dismutase (*SOD1*) in rodents has provided useful models for studying the pathogenesis of amyotrophic lateral sclerosis (ALS). Microglia have been shown to contribute to ALS disease progression in these models, although the mechanism of this contribution remains to be elucidated. Here, we present the first evidence of the effects of overexpression of mutant (TG G93A) and wild type (TG WT) human *SOD1 *transgenes on a set of functional properties of microglia relevant to ALS progression, including expression of integrin β-1, spreading and migration, phagocytosis of apoptotic neuronal cell debris, and intracellular calcium changes in response to an inflammatory stimulus.

**Results:**

TG SOD1 G93A but not TG SOD1 WT microglia had lower expression levels of the cell adhesion molecule subunit integrin β-1 than their NTG control cells [NTG (G93A) and NTG (WT), respectively, 92.8 ± 2.8% on TG G93A, 92.0 ± 6.6% on TG WT, 100.0 ± 1.6% on NTG (G93A), and 100.0 ± 2.7% on NTG (WT) cells], resulting in decreased spreading ability, with no effect on ability to migrate. Both TG G93A and TG WT microglia had reduced capacity to phagocytose apoptotic neuronal cell debris (13.0 ± 1.3% for TG G93A, 16.5 ± 1.9% for TG WT, 28.6 ± 1.8% for NTG (G93A), and 26.9 ± 2.8% for NTG (WT) cells). Extracellular stimulation of microglia with ATP resulted in smaller increase in intracellular free calcium in TG G93A and TG WT microglia relative to NTG controls (0.28 ± 0.02 μM for TG G93A, 0.24 ± 0.03 μM for TG WT, 0.39 ± 0.03 μM for NTG (G93A), and 0.37 ± 0.05 μM for NTG (WT) microglia).

**Conclusions:**

These findings indicate that, under resting conditions, microglia from mutant *SOD1 *transgenic mice have a reduced capacity to elicit physiological responses following tissue disturbances and that higher levels of stimulatory signals, and/or prolonged stimulation may be necessary to initiate these responses. Overall, resting mutant *SOD1*-overexpressing microglia may have reduced capacity to function as sensors of disturbed tissue/cellular homeostasis in the CNS and thus have reduced neuroprotective function.

## Background

Amyotrophic lateral sclerosis (ALS) is a progressive neurodegenerative disorder characterized by selective demise of upper motor neurons in the motor cortex and lower motor neurons in the brainstem and spinal cord [1,2]. Disease onset occurs in mid-life (50 to 60 years of age) and is followed by a rapid (2 to 5 years), progressive failure of the neuromuscular system and death. Although the aetiology of ALS is yet to be fully elucidated, several factors are likely to contribute to motor neuron injury, including excitotoxic and oxidative motor neuron damage, protein aggregation, impaired axonal transport, mitochondrial dysfunction, and non-cell autonomous damage mediated through glial cells - astrocytes and microglia [3,4]. Most of the current insights into disease pathogenesis come from studies on animal models overexpressing mutant forms of Cu/Zn superoxide dismutase 1 (*SOD1*) [5]. Autosomal dominant inheritance of mutant *SOD1 *accounts for 20 percent of familial ALS (FALS) cases, or 2 percent of all ALS cases [6,7]. Overexpression of mutant forms of SOD1, including G93A, G37R and G85R mutant SOD1, in animal models faithfully replicates pathological features of the human disease [8-10]. Motor neurons expressing mutant *SOD1 *can escape disease if surrounded by a sufficient number of normal non-neuronal cells [11]. Conversely, normal motor neurons surrounded by mutant SOD1-containing non-neuronal cells developed signs of cellular injury with the development of ubiquitinated protein deposits [11]. Selectively reducing the levels of mutant *SOD1 *in motor neurons delayed early disease progression and extended lifespan by a mean of 22 percent (64 days). In contrast, reducing mutant *SOD1 *expression in microglia, the major immune cell of the CNS with a monocyte/macrophage phenotype, had no effect on onset and early disease but showed a large protective effect in late stage disease and ameliorated disease progression with a mean extension of survival of 99 days [12]. Moreover, a significant slowing of disease progression was observed in double transgenic G93A-SOD1/PU.1^-/- ^mice when the mice received wild type but not G93A-SOD1 bone marrow transplant [13].

While the mechanisms of microglial disease propagation remain to be fully elucidated, studies indicate that mutant *SOD1*-overexpressing microglia may acquire an exaggerated inflammatory phenotype and neurotoxic properties following sustained activation. Low levels of inflammatory mediators are present in the cerebrospinal fluid of ALS patients [14-16] and activated microglia are detected in the CNS [17] and in the neighbourhood of degenerating motor neurons in post-mortem studies of the human disease [18]. *SOD1 *transgenic mouse and rat models of ALS also display signs of an inflammatory response in the CNS at all stages of the disease. Prior to the clinical signs of disease onset, microglia are in an early state of activation, and elevated levels of inflammatory mediators such as interleukin (IL)-6 can be detected [19,20]. With the onset of symptoms and motor neuron cell death, fully activated (or reactive) microglia are present in the CNS and microglial production of the pro-inflammatory cytokine, tumour necrosis factor (TNF)-α has been demonstrated [21-24]. Elevated levels of TNF-α, monocyte chemoattractant protein (MCP)-1, macrophage-colony stimulating factor (M-CSF), interferon (IFN)-γ and transforming growth factor (TGF)-β [15,23,25,26] and an increase in cyclooxygenase (COX)-2 activity and prostaglandin (PG) E2 levels [14,15,23,25,27] have been shown in mutant *SOD1 *transgenic mouse tissues and microglial cells. Administration of drugs such as minocycline, or inhibitors of COX-2 and peroxisome proliferator-activated receptor (PPAR), capable of reducing microglial activation, delayed both disease onset and progression in mutant *SOD1 *transgenic mice [28-31].

It is unknown whether microglia overexpressing the wild type form of human *SOD1 *can acquire altered functional properties. Overexpression of wild type *SOD1 *in animals did not reveal any overt pathology at four months of age [32] except signs of deficiency of muscle innervation and premature aging [33-36]. Thus, wild type SOD1 could also contribute to neuronal pathogenesis. For example, autopsy material from familial as well as sporadic ALS cases revealed Lewy body-like hyaline inclusions within motor neurons that immunoreacted with anti-SOD1 antibodies [37]. Overexpression of wild type *SOD1 *in mutant *SOD1 *transgenic animals accelerated the disease course and shortened the lifespan of double transgenic animals [38]. Additionally, wild type SOD1 acquired toxic properties similar to those of the mutant forms of SOD1 following oxidative damage [39]. Therefore, it is possible that wild type *SOD1*-overexpressing microglia may also have altered cellular properties rendering the cells capable of propagating neuronal damage.

In healthy animals, microglia perform a surveillance function to maintain a physiologically healthy microenvironment [40]. They accomplish this by sampling the surrounding tissue with numerous extruding and retracting processes [41]. Alterations in the tissue microenvironment induce microglial migration to the site of damage, scavenging of extruded cellular or plasma proteins and clearance of damaged cell components through phagocytosis [41,42]. These dynamic effector functions of microglia are dependent on the presence of diverse surface receptors, including cytokine, chemokine, immunoglobulin, and purinergic receptors [43,44]. Efficient intracellular signalling, control of gene expression, and tightly regulated function of the actin cytoskeleton are also necessary for appropriate microglial effector responses [45,46]. Interestingly, *in vivo *recordings of labelled microglia from *SOD1 G93A*-overexpressing mice revealed significantly increased microglial response towards laser-induced single axon transection at preclinical age (60 days) when compared to that in control mice, and a subsequent reduction in SOD1 G93A microglial response to the same injury with disease progression (90 and 120 days) [47].

The purpose of the current study was to investigate whether overexpression of the mutant *SOD1 *transgene (TG G93A) or the wild type *SOD1 *transgene (TG WT) in microglia could significantly alter their functional properties, potentially contributing to neurodegeneration and its propagation. Due to inherent differences between the two colonies of transgenic mice that we observed in our studies, we compared the differences between NTG and TG cells within colonies, and not between colonies. Specifically, we examined non-transgenic (NTG: NTG (G93A) and NTG (WT)) and *SOD1*-overexpressing transgenic (TG G93A and TG WT) microglial surface expression of integrin β-1 (a subunit of integrin cell adhesion molecules), the ability of microglia to spread on fibronectin-coated surfaces and to migrate over astrocytic monolayers, the ability to phagocytose apoptotic neuronal cell debris, and intracellular calcium changes in response to a pro-inflammatory stimulus, extracellular ATP. Mutant SOD1 caused the most marked changes in these functions but overexpression of wild type SOD1 also produced significant changes. Thus, it is essential to examine the effects of both mutant and wild-type SOD1 when investigating the role of microglial cells in ALS.

## Results

### Reduced expression of integrin β-1 by TG G93A microglia

**Figure 1:**
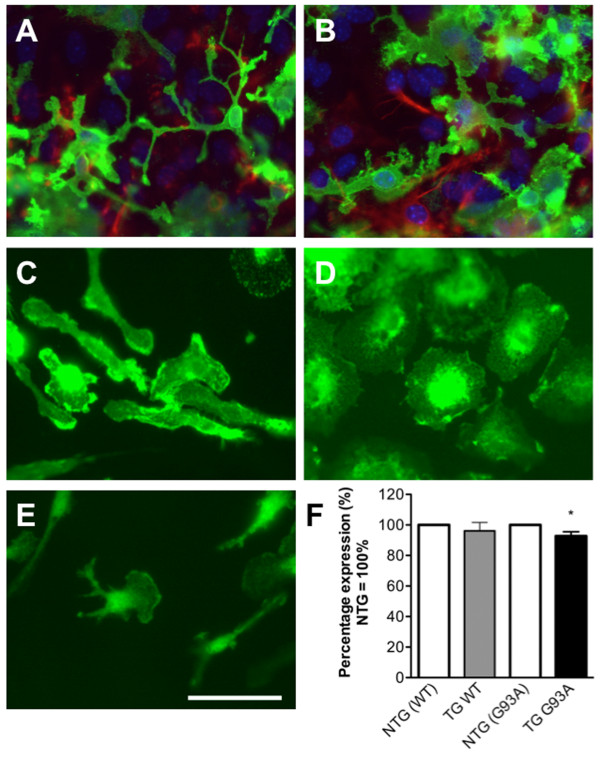
**Microglial culture purity and reduced expression of integrin β-1 by TG G93A microglia**. (A and B) Immunostaining of representative mixed glial cultures used to purify microglia. The cultures were stained with anti-CD11b (green) and anti-GFAP (red) antibodies to show microglia in green and astrocytes in red; nuclei were stained with Hoechst (blue). (C and D) Immunostaining with anti-F4/80 antibody of purified resting (C) and activated with lipopolysaccharide (D) microglia. (E) Immunostaining of purified microglia with isotype control antibody shows residual non-specific fluorescence with the isotype-matched antibody. Scale bar = 50 μm. (F) Cell surface integrin β-1 expression levels on NTG (WT), TG WT, NTG (G93A) and TG G93A microglia. Data are presented as mean ± SEM; Student's t test, * p = 0.0493 versus NTG (G93A) cells, n = 14 TG G93A and nine NTG (G93A) mice from four litters, five TG WT and five NTG (WT) mice from two litters.

Microglia were purified from mixed glial cultures of TG WT, NTG (WT), TG G93A and NTG (G93A) mice. Figure 1 presents immunostaining images of microglia in mixed glial cultures and following purification. Mixed glial cultures contained astrocytes and microglia (Figure 1A and 1B) but purification using a mild trypsinisation method resulted in cultures consistent of cells positive for the monocyte/macrophage marker F4/80 - microglia (Figure 1C and 1D). To investigate if overexpression of *SOD1 *in microglia could alter their interaction with the extracellular matrix, cell surface expression of integrin β-1 (the β1 subunit of integrins, a fibronectin receptor) was measured in TG G93A and TG WT microglia and compared to those in respective NTG cells. While TG WT microglia had integrin β-1 levels comparable to those in NTG (WT) cells (mean ± standard error of the mean (SEM): 91.96 ± 6.6% on TG WT versus 100 ± 2.7% on NTG (WT) cells; Figure 1F), the expression of integrin β-1 on TG G93A microglia was modestly but significantly reduced when compared to NTG (G93A) cells (mean ± SEM: 92.76 ± 2.8% on TG G93A versus 100 ± 1.6% on NTG (G93A) cells; p = 0.0493, Student's t test, Figure 1F). Additional file 1 presents the data of Figure 1F in more detail.

### Reduced spreading ability of TG G93A microglia

**Figure 2:**
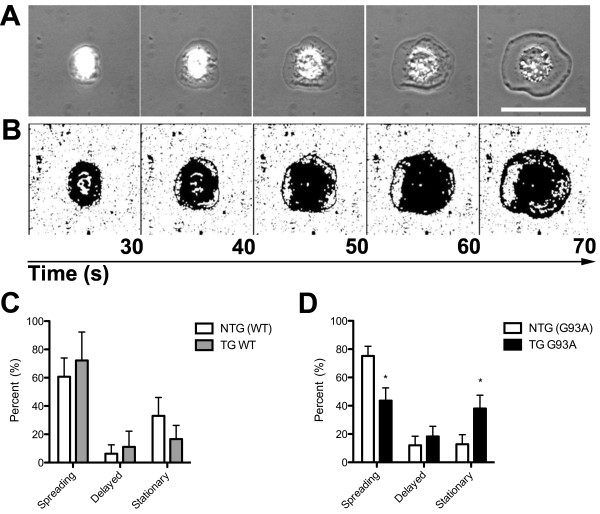
**Reduced spreading ability of TG G93A microglia**. (A) Representative time-lapse frames of a microglial cell spreading on a fibronectin-coated surface are shown as an example. Scale bar = 10 μm. (B) The same frames as in A modified for analysis of cell surface area. The frames are matched with the time (in seconds (s) indicated above arrow) that elapsed from the initiation of recording. (C) Total spreading profile of NTG (WT) and TG WT microglia. Microglia that initiated spreading within first 100 s of recording were classified as "Spreading". Microglia that initiated spreading after the first 100 s of recording were classified as "Delayed". Microglia that did not spread during the entire recording time were classified as "Stationary". (D) Total spreading profile of NTG (G93A) and TG G93A microglia. Data are mean ± SEM; Student's t test, * p = 0.0195 for Spreading and * p = 0.0483 for Stationary TG G93A microglia versus respective NTG (G93A) cells, n = ten TG G93A mice (36 cells) and nine NTG (G93A) mice (30 cells) from four litters, and three TG WT mice (14 cells) and four NTG (WT) mice (14 cells) from two litters.

To investigate whether the reduced expression levels of integrin β-1 by TG G93A microglia could impair interactions of TG G93A microglia with the extracellular matrix, the spreading of microglial cells on a fibronectin-coated surface was examined. Figure 2 shows representative images captured for a spreading microglial cell (Figure 2A), and the same images modified for cell surface area calculations (Figure 2B). Individual microglia were analysed for the time taken to initiate spreading after attaching to the fibronectin-coated surface, and the subsequent speed of spreading, if initiation was successful. The spreading profiles of TG WT and NTG (WT) microglia were similar, and consisted of comparable percentages of cells that spread immediately, after a delay of 100 s before initiating spreading, or that were stationary, i.e. that failed to spread (immediate: 72.2 ± 20.0% in TG WT versus 60.7 ± 13.0% in NTG (WT) cells; delayed: 11.1 ± 11.1% in TG WT versus 6.3 ± 6.3% in NTG (WT) cells; stationary: 16.7 ± 9.6% in TG WT versus 33.0 ± 13.0% in NTG (WT) cells, Figure 2C). In contrast, the spreading profiles of TG G93A and NTG (G93A) microglia differed significantly. TG G93A microglia had a lower percentage of immediate and a higher percentage of stationary cells when compared to NTG (G93A) microglia (immediate: 43.7 ± 8.9% in TG G93A versus 73.5 ± 7.1% in NTG (G93A) cells; p = 0.0195, Student's t test; delayed: 18.3 ± 7.1% in TG G93A versus 11.5 ± 6.3% in NTG (G93A) cells; stationary: 38.0 ± 9.5% in TG G93A versus 12.8 ± 6.7% in NTG (G93A) cells; p = 0.0483, Student's t test, Figure 2D).

### Spreading and migration speeds are unaltered in TG G93A microglia but are increased in TG WT cells

**Figure 3:**
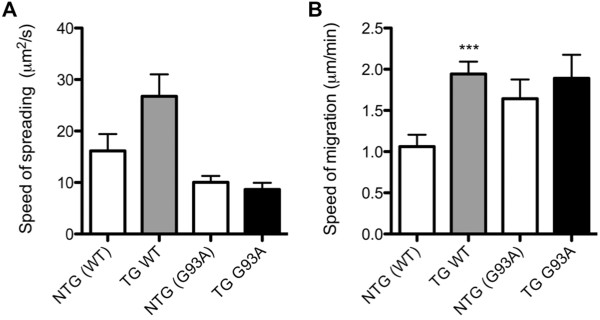
**Spreading and migration speeds of TG G93A and TG WT microglia**. (A) Speed of spreading (μm^2^/s) of NTG (WT), TG WT, NTG (G93A) and TG G93A microglia, n = three TG WT mice (12 cells) and three NTG (WT) mice (ten cells) obtained from two litters, and ten TG G93A mice (15 cells) and nine NTG (G93A) mice (20 cells) from four litters. (B) Speed of migration (μm/min) of NTG (WT), TG WT, NTG (G93A) and TG G93A microglia on genotype-matched astrocytic monolayers. Data are mean ± SEM; Student's t test, *** p = 0.0004 versus NTG (WT) microglia, n = three TG WT mice (ten cells) and three NTG (WT) mice (ten cells) from two litters.

To further investigate if the reduced expression levels of integrin β-1 by TG G93A microglia affected microglial spreading and migration, the speeds of spreading and migration were measured. While the ability to spread on fibronectin-coated surface was impaired in the TG G93A microglia (Figure 2D), the speed of spreading of those TG G93A microglia that did spread was not different from that of spreading NTG (G93A) microglia (8.6 ± 1.3 μm^2^/s for TG G93A versus 10 ± 1.3 μm^2^/s for NTG (G93A) microglia, Figure 3A). Interestingly, TG WT microglia that did spread had a greater speed of spreading compared to NTG (WT), albeit not statistically significant (26.6 ± 4.3 μm^2^/s for TG WT versus 16.2 ± 3.3 μm^2^/s for NTG (WT) microglia; p = 0.0693, Student's t test, Figure 3A). To investigate if the spreading impairment of TG G93A microglia could affect migration, the speed of random migration of NTG (G93A), TG G93A, NTG (WT) and TG WT microglia on genotype-matched astrocytic monolayers was measured. TG G93A microglia had similar speed of migration as NTG (G93A) microglia (1.9 ± 0.3 μm/min for TG G93A versus 1.6 ± 0.2 μm/min for NTG (G93A) microglia). Again, TG WT cells showed increased speed of migration when compared to NTG (WT) microglia (1.9 ± 0.1 μm/min for TG WT versus 1.1 ± 0.1 μm/min for NTG (WT) cells, p = 0.0004, Student's t test, Figure 3B). Directional migration of NTG (G93A), TG G93A, NTG (WT) and TG WT microglia towards the chemoattractant MCP-1 was also investigated (Additional file 2), without detectable differences in distance migrated between the cells of the four genotypes (data not shown).

### Reduced ability of TG WT and TG G93A microglia to phagocytose apoptotic neuronal cell debris

**Figure 4:**
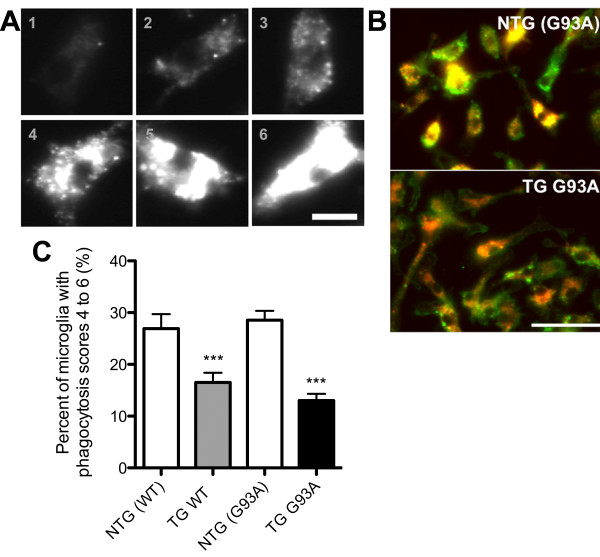
**Reduced potential of TG WT and TG G93A microglia to phagocytose apoptotic neuronal cell debris**. (A) Scoring scale (from 1 to 6) used to measure microglial phagocytic potential. Bar = 10 μm. (B) Representative images of NTG (G93A) and TG G93A microglia immunostained with anti-integrin β-1 antibody (green) after incubation with VybrantDiI-labelled apoptotic neuronal cell debris (red-orange). Scale bar = 50 μm. (C) Percentage of microglia with moderate to high phagocytic potential (scores 4 to 6). Data are mean ± SEM; Student's t test, *** p = 0.0001 versus respective NTG cells, n = six NTG (WT) and seven TG WT mice from three litters, and six NTG (G93A) and six TG G93A mice from two litters, capturing 10-12 images per mouse.

To establish if the overexpression of *SOD1 *in microglia altered the ability to phagocytose apoptotic cell debris, the phagocytosis of apoptotic murine neuronal cell (NSC34) debris by microglia was investigated. Microglial cells, incubated with apoptotic NSC34 neuronal cell debris, labelled with a membrane dye VybrantDiI, were extensively washed to remove any unphagocytosed material and immunostained with anti-integrin β-1 antibody to delineate the plasma membrane. Figure 4 presents the scoring scale used to score the level of phagocytosis (Figure 4A) and representative immunostaining pictures of NTG (G93A) and TG G93A microglia with the phagocytosed material (Figure 4B). The percentage of microglia with medium (score 4) to high (score 6) loads of phagocytic material were compared between the four genotypes. TG G93A and TG WT microglia had a significantly lower percentage of microglia scoring from 4 to 6 than the respective NTG cells (13.0 ± 1.3% for TG G93A, 16.5 ± 1.9% for TG WT, 28.6 ± 1.8% for NTG (G93A) and 26.9 ± 2.8% for NTG (WT) microglia, p = 0.0001, Student's t test, Figure 4C).

### Reduced intracellular calcium release in TG WT and TG G93A microglia following extracellular ATP stimulation

**Figure 5:**
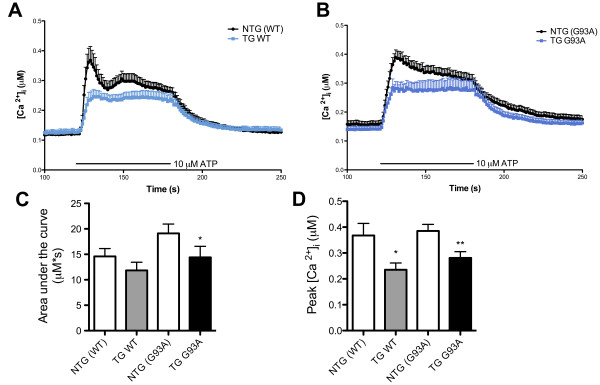
**Reduced intracellular calcium release in TG WT and TG G93A microglia following extracellular ATP stimulation**. (A) Intracellular calcium concentration ([Ca^2+^]_i_, μM) changes in NTG (WT) and TG WT microglia following extracellular stimulation with 10 μM ATP for 1 min. The period of ATP challenge is indicated with the bar. (B) Changes of [Ca^2+^]_i _in NTG (G93A) and TG G93A microglia following extracellular stimulation with 10 μM ATP for 1 min. The period of ATP challenge is indicated with the bar. (C) Area-under-the-curve (AUC) values (μM*s) for NTG (WT), TG WT, NTG (G93A) and TG G93A microglia. Data are mean ± SEM; Student's t test, *p = 0.0176 versus NTG (G93A) microglia. (D) Highest [Ca^2+^]_i _values (μM) recorded for NTG (WT), TG WT, NTG (G93A) and TG G93A microglia during 1 min challenge with 10 μM ATP. Data are mean ± SEM; Student's t test, *p = 0.0123 versus NTG (WT) and **p = 0.003 versus NTG (G93A) cells, n = three NTG (WT) mice (38 cells) and three TG WT mice (39 cells) from two litters, three NTG (G93A) mice (50 cells) and three TG G93A mice (40 cells) from two litters.

To investigate whether the overexpression of *SOD1 *in microglia affected intracellular calcium changes following a pro-inflammatory stimulus, microglial intracellular calcium concentration ([Ca^2+^]_i_) was measured before, during, and after stimulation with 10 μM ATP. Stimulation with ATP increased [Ca^2+^]_i _and withdrawal of ATP resulted in a steady reduction of the [Ca^2+^]_i _to almost baseline levels in microglia of all genotypes (Figure 5A and 5B). Microglia of all four genotypes had comparable baseline [Ca^2+^]_i_: 0.16 ± 0.01 μM for NTG (G93A), 0.14 ± 0.01 μM for TG G93A, 0.12 ± 0.01 μM for NTG (WT) and 0.13 ± 0.01 μM for TG WT. The first 10 s of ATP stimulation resulted in an initial rapid increase of [Ca^2+^]_i _in NTG microglia, followed by a steady reduction of [Ca^2+^]_i _during the remaining 50 s of ATP challenge. However, TG microglia displayed a different pattern of [Ca^2+^]_i _changes; the initial peak in [Ca^2+^]_i _was absent in both TG G93A and TG WT microglia (Figure 5A and 5B). The first 10 s of ATP challenge only moderately increased the [Ca^2+^]_i _in TG G93A and TG WT microglia. This [Ca^2+^]_i _was maintained until ATP was withdrawn. The area-under-the-curve (AUC) calculations quantified this pattern of [Ca^2+^]_i _changes, and showed areas of 19.1 ± 1.8 μM*s for NTG (G93A) versus 14.4 ± 2.2 μM*s for TG G93A microglia, p = 0.0176, Student's t test, and 14.6 ± 1.5 μM*s for NTG (WT) versus 11.9 ± 1.6 μM*s for TG WT cells, not significantly different (Figure 5C). The highest value of [Ca^2+^]_i _was significantly smaller in TG G93A and TG WT microglia when compared to respective NTG cells and was 0.28 ± 0.02 μM for TG G93A versus 0.39 ± 0.03 μM for NTG (G93A) cells, p = 0.003, Student's t test, and 0.24 ± 0.03 μM for TG WT versus 0.37 ± 0.05 μM for NTG (WT) microglia, p = 0.0123, Student's t test (Figure 5D). Interestingly, stimulation of microglia with a high concentration of ATP (1 mM for 1 min) reversed this initial reduced response in [Ca^2+^]_i _changes, but only in TG G93A microglia (Additional file 3).

## Discussion

In the present study we compared an array of functional properties of transgenic microglia overexpressing wild type *SOD1 *or mutant *SOD1 G93A *to those of non-transgenic control cells.

Integrin β-1 (fibronectin receptor β) is involved in diverse cellular functions including cellular adhesion to extracellular matrix, focal adhesion, migration, actin polymerisation/assembly, and regulation of the cell cycle [48-50]. We observed only modestly reduced levels of integrin β-1 on TG G93A microglia when compared to the levels on NTG (G93A) cells. Also, the small sample size of TG WT and NTG (WT) microglia may have prevented the detection of differences in integrin β-1 expression levels in this cell population. Nonetheless, reduced levels of integrin β-1 on TG G93A microglia could lead to impaired adhesion, migration, phagocytosis, and proliferation. To test whether the former three functions were altered in TG G93A microglia, we examined microglial spreading on fibronectin-coated surface, migration on genotype-matched astrocytic monolayers, and phagocytosis of apoptotic neuronal cell debris. We observed that although expression of integrin β-1 was marginally reduced, and spreading ability impaired, in TG G93A cells, these changes did not affect the migration of TG G93A microglia, as the cells were capable of migrating on genotype-matched astrocytes at the same speed as NTG (G93A) cells. This observation may indicate that the slightly reduced levels of integrin β-1 expression on TG G93A microglia may have no significant impact on the cellular spreading and migration, or that additional receptor-ligand interactions compensate for reduced integrin β-1 expression and impaired spreading ability.

Our study also showed that overexpression of both transgenes affected microglial phagocytosis of apoptotic neuronal cell debris, an effect presumably unrelated to the reduced integrin β-1 expression. Microglial uptake of degenerating motor neuronal debris has been shown *in vivo *after injection of toxin ricin into rat facial nerve [51]. Microglia were the primary cells to clear neuronal debris from degenerating neurons during development or after neuronal injury in adult animals [52,53]. Efficient clearance of SOD1 aggregates significantly improved the disease course in ALS model mice [54]. Moreover, persistence of destroyed tissue debris was capable of inducing and perpetuating CNS tissue inflammation [55,56]. Thus, the impaired ability to phagocytose apoptotic neuronal debris by TG WT, and more so by TG G93A, microglia may contribute to disease exacerbation.

Overexpression of *SOD1 G93A *as well as wild type *SOD1 *altered the intracellular Ca^2+ ^responses to extracellular ATP stimulation. Cellular responses to extracellular nucleotides are mediated via cell surface P2 purinoreceptors, now classified as metabotropic (P2Y) receptors that mediate signalling through G-proteins, and ionotropic (P2X) receptors that are directly coupled to non-selective cationic receptors allowing for influx of Ca^2+ ^and Na^+ ^and efflux of K^+ ^[57]. Physiologically, ATP-induced signalling attracts microglia to the site of injury or cellular damage resulting in microglial activation [42,58]. Microglia express P2X_4_, P2X_7_, P2Y_1_, P2Y_2_, P2Y_6 _and P2Y_12 _receptors [59-62], and their stimulation initiates release of Ca^2+ ^from intracellular stores. In addition, stimulation through P2X receptors induces non-selective ion exchange that may result is microglial depolarisation [63]. Microglia from *SOD1 G93A*-overexpressing ALS mouse models were found to have an increased expression of P2X_4_, P2X_7 _and P2Y_6 _receptors and reduced ability to hydrolyse extracellular ATP [64]. Moreover, prolonged (3 to 24 hours) stimulation with 30 μM ATP induced higher levels of COX-2 expression and elevated production of the pro-inflammatory cytokine TNF-α in SOD1 G93A microglia compared to NTG cells [64], demonstrating the exaggerated pro-inflammatory phenotype of SOD1 G93A microglia. However, during our experiments, short (1 min) stimulation of microglia with 10 μM ATP demonstrated that microglia overexpressing *SOD1 G93A*, or wild type *SOD1*, had a reduced initial increase in intracellular Ca^2+ ^when compared to that in NTG cells, which suggests an alteration of calcium release from the endoplasmic reticulum. Thus, higher ATP levels, and/or prolonged stimulation by the nucleotide, may be necessary in order to elicit physiologically relevant responses from *SOD1*-overexpressing microglia, although overstimulation leads to exaggerated pro-inflammatory response from TG G93A microglia (Reference [26] and Additional file 3).

To summarise, it is well characterized that in ALS animal models activated microglia contribute to neurodegeneration through production of neurotoxic compounds following activation [13,64] and acquisition of an exaggerated inflammatory phenotype [24,26]. Here, we show evidence that *SOD1 *transgenic microglia have a reduced capacity to sense tissue disturbances under resting conditions. The affected functions in resting SOD1 microglia are a reduced ability to phagocytose apoptotic neuronal cell debris and an attenuated response to extracellular ATP stimulation. We propose that resting state microglia overexpressing *SOD1 G93A*, or wild type *SOD1*, may contribute to disease pathogenesis through loss of efficient tissue-protective functions. Also, caution must be used when ascribing alterations in microglial behaviour to mutant *SOD1 *expression. Our results indicate that wild-type *SOD1 *overexpression can alter some properties of microglia and should be an obligatory control in all experiments.

## Conclusions

Microglia, the macrophages of the brain, are known to play vital physiological roles in maintaining healthy CNS tissue architecture and function. In ALS pathogenesis, microglia contribute to disease progression through continuous activation and secretion of multiple pro-inflammatory and neurotoxic mediators. Here, we present the novel findings that resting-state microglia overexpressing wild type or mutant *SOD1 *transgenes have reduced physiologic responses of apoptotic neuronal cell clearance and release of Ca^2+ ^from intracellular stores upon ATP stimulation. Our findings indicate that transgenic *SOD1 *microglia may require higher concentrations of stimulatory factors to elicit physiologically-relevant functions, indicating reduced neuroprotective behaviour of TG microglia.

## Methods

### Primary microglial culture preparation

All animals were handled in accordance with the guidelines of the Animals (Scientific Procedures) Act 1986. Microglia were purified from mixed glial cultures as described [26]. Briefly, the cortices of neonatal (1-2 days old) human mutant *SOD1 G93A *transgenic mice, human wild type *SOD1 *transgenic mice, and their nontransgenic (NTG) littermates (NTG (G93A) and NTG (WT), respectively) were stripped of meninges, washed and triturated in Hank's balanced salt solution with Ca^2+^/Mg^2+ ^containing 0.04% trypsin (Sigma), 0.1 mg/ml collagenase (Calbiochem) and 0.05 mg/ml DNaseI (Sigma). After trituration, the single cells were pelleted and plated in complete medium [Dulbecco's modified Eagle's medium (DMEM; Cambrex Bioscience), 10% heat-inactivated foetal calf serum (FCS; BioSera), 100 units/ml penicillin and 100 mg/ml streptomycin (Gibco, Invitrogen)] at 60 000 cells/cm^2 ^on poly-L-lysine (Sigma) coated coverslips. For the purification of microglia, the confluent cultures were subjected to shaking and mild trypsinisation [13,65], which resulted in microglial cultures of more than 90% purity.

### Immunostaining

The cells were washed with phosphate-buffered saline (PBS), fixed with 4% paraformaldehyde for 15 min and permeabilised with 0.1% Triton X-100. Non-specific binding was blocked with 5% FCS in PBS for 30 min. The cells were incubated with rat anti-mouse CD11b (Serotec), rat anti-mouse F4/80 (Serotec), or isotype control (Serotec) primary antibodies in blocking buffer at room temperature. After washing with PBS, cells were incubated with the goat anti-rat-fluorescein isothiocyanate (FITC) secondary antibody (Serotec) and, where needed, with conjugated Cy3-anti-glial fibrillary acidic protein (GFAP) antibody (Sigma) in blocking buffer. After washing, the nuclei were stained with 0.2 μg/ml Hoechst 33342 (Intergen) solution for 1 min and the coverslips mounted on glass sides in mounting medium (50% glycerol in PBS).

### Measurement of integrin β-1 expression

Microglia isolated from mixed glial cultures were deposited into U-bottomed 96-well plates at 20 000 cells/well, washed in 200 μl fluorescence activated cell sorting (FACS) buffer (PBS containing 0.2% w/v bovine serum albumin (BSA) and 0.1% w/v sodium azide) and incubated with conjugated FITC-anti-mouse integrin β-1 (Biolegend) or FITC-isotype control antibody (Biolegend) in FACS buffer for 20 min on ice. Cell-associated mean fluorescence intensity (MFI) was detected using a BD FACSort (Becton Dickinson). The acquired geometric mean fluorescence values of isotype antibody-stained cells were subtracted from those of the FITC-integrin β-1 stained cells. The resultant values were then compared between respective NTG and TG cells, after counting the mean MFI of NTG cells as 100% (Additional file 1).

### Spreading assay

Isolated microglia were added to an imaging chamber containing a coverslip coated with 5 μg/ml of bovine fibronectin (Calbiochem) as a substrate. Cells were visualized under a × 40 oil immersion objective heated to 37°C with an objective heater (Bioptechs, Intracel) and the spreading of individual cells in phase contrast recorded by a time-lapse automation, using Openlab 3.7.1 software. After the cells touched the coated coverslip, the frames were captured every 5 s for a maximum of 15 min. The captured frames were analysed with ImageJ plugins to obtain cell surface area values for every frame. The cells were classified as "spreading" if they initiated cell surface area expansion (spreading) within first 100 s of recording. The cells were classified as "delayed" if they initiated cell surface area expansion after the first 100 s of recording. The cells were classified as "stationary" if they did not initiate cell surface area increase during the entire recording time. For every spreading cell, the cell surface area values of the expanding (spreading) phase were plotted against the time taken to reach those values, and the speed of spreading was deduced from this relationship using GraphPad Prism 5.0 linear regression analysis.

### Migration assay

Mixed glial cultures in T25 flasks (Croning) were placed in a microscope chamber equilibrated to 37°C, 5% CO_2_, (LEICA Microsystems AF6000LX microscope with environmental control) for time-lapse recordings. A × 10 objective field of cells was captured every 3 min for 15 h. Using phase contrast microscopy, small phase-bright amoeboid and ramified microglia were easily visible on top of the larger astrocytes that formed a confluent monolayer. The captured frames were loaded into ImageJ software as one stack, and microglia were chosen at random from each field of view and analysed for the distance migrated.

### Phagocytosis of neuronal cell debris

NSC34 cells (a murine motor neuronal cell line [66]) were labelled with a membrane dye [1, 1'-dioctadecyl-3,3,3',3'-tetramethylindocarboxyanine perchlorate (VybrantDiI) with an emission wavelength in red-orange spectrum (Invitrogen)] according to the manufacturer's protocol. The cells were then incubated for 48 h in serum free DMEM to induce apoptosis through oxidative stress [67], at which point cell death had occurred in 90% of cells. The labelled NSC34 cell debris was collected and frozen at -80°C. Microglial cells plated on poly-L-lysine (Sigma) -coated 13 mm coverslips were incubated with 500 μl of labelled NSC34 cell debris for 21 h at 37°C. Microglia were extensively washed with PBS to remove any unphagocytosed debris, and immunostained with conjugated FITC-anti-mouse integrin β-1 (Biolegend) antibody. Using fluorescence microscopy, an operator blinded to the genotypes of microglial cells scored the cells according to the uptake of VybrantDiI-labelled material on a scale from 1 to 6 (Figure 4A).

### Measurement of intracellular calcium concentration

The measurements of microglial intracellular free calcium were carried out according to a previously published method [68,69]. The cells were pre-incubated with the membrane-permeable ester form of the high-affinity ratiometric calcium dye Fura-2 AM (10 μM) for 15 min and allowed to de-esterify for 30 min at room temperature (25°C). The cells were placed in a recording chamber (3 ml), which was continuously perfused (10 ml/min) with a standard extracellular solution containing: HEPES 11.6 mM, Na^+^129.1 mM, Cl^- ^143.8 mM, K^+ ^5.9 mM, Mg^2+ ^1.2 mM, Ca^2+ ^3.2 mM, and glucose 10.0 mM, at pH = 7.3. Fluorescent images were obtained using × 40 objective on an Axiovert 200 microscope (Carl Zeiss) fitted with a C9100 electron multiplier CCD camera (Hamamatsu Photonics) at 345/380 nm excitation (Polychrome IV, Till Photonics) and 510 nm emission filters. Devices and shutters were controlled by Openlab 3.7.1 software, which provided image acquisition and continuous online Ca^2+ ^concentration calculation by custom scripting at 1/s. The regions of interest were defined over perinuclear/cytoplasmic regions of microglia and changes in fluorescence ratios (345/380) recorded at rest and during stimulation with specified concentrations of ATP, which was applied with a custom-made solution applicator with a solution exchange time of ~100 ms in the area of view. Background fluorescence subtraction was used and calcium concentration calculated according to Grynkiewicz [70].

### Statistical analysis

Due to inherent differences between the two transgenic colonies of mice (transgenic *SOD1 G93A *and transgenic *SOD1 *wild type) that we observed in our past experiments, we limited our statistical comparisons to measure differences between NTG and TG cells within colonies, and not between colonies. Data were analysed using GraphPad Prism 5.0 software. All data that fitted a normal distribution were analysed using Student's t test. Data that did not fit a normal distribution were transformed using Y = Log(Y) transformation.

## Competing interests

The authors declare that they have no competing interests.

## Authors' contributions

SAS performed the immunostaining, flow cytometry, spreading, and calcium concentration measurement studies, as well as assisted with the manuscript preparation. DJB performed the migration assays and contributed to the manuscript preparation. SCB helped with the statistical analysis and manuscript preparation. KJDV designed the spreading assay, wrote the Openlab 3.7.1 automation for time-lapse imaging, and assisted with manuscript preparation. JG developed the assay for ATP stimulation and intracellular calcium concentration measurements and the analysis of the obtained results, and helped with the manuscript preparation. PJS was the grant holder and PNM and PJS supervised the study and prepared the manuscript. All authors read and approved the final manuscript.

## Supplementary Material

Additional file 1 **Calculation of percent expression of integrin β-1 on primary microglia**. (A) A representative example of flow cytometry histograms for NTG (G93A) and TG G93A microglia with FITC-isotype antibody or FITC-anti-integrin β-1 antibody is presented. Each histogram contains data on the geometric mean fluorescence (Gm), coefficient of variance (CV), marker position (0-430), number and percentage of cells under the marker. (B) An overlay of histograms from another independent experiment represents the FITC signal distribution for FITC-isotype antibody stained TG G93A microglia (filled curve), FITC-anti-integrin β-1 antibody stained TG G93A microglia (curve with dashed line) and FITC-anti-integrin β-1 antibody stained NTG (G93A) microglia (curve with solid line). The horizontal axis in (A) and (B), FL-1-H, is the detected signal intensity of FITC on a logarithmic scale. An example of percentage expression calculation is given below: 1. Under the marker M1 over 95% of the cells are detected, with the Gm for NTG (G93A) microglia of 11.11 with specific antibody, and 4.54 with isotype antibody. 2. For these NTG (G93A) microglia the integrin β-1 expression is 11.11 - 4.54 = **6.57**, and for TG G93A cells the integrin β-1 expression is 10.62 - 4.93 = **5.69**. These expression values are then converted to values on a linear scale. The values for NTG (G93A) are set as 100% expression. The values from TG (G93A) then converted to percent expression with respect to 100% of NTG (G93A) cells. Click here for file

Additional file 2 **Migration of TG WT and TG G93A microglia towards the chemoattractant MCP-1: **(A) A representative image of microglial cells on fibronectin-coated coverglass overlaying a Dunn migration chamber with cells facing the chamber. The image was taken on the LEICA Microsystems AF6000LX microscope on a x10 objective, with environmental control to maintain the slide at 37°C. To record migration, images were taken every 3 minutes for 1 hour using Leica software. At the top of the picture is the edge of the outer well, which was filled with medium containing MCP-1. The lower edge is the inner well containing normal medium. Scale bar = 50 μm. (B) An example of analysis of migrated distance represents vector diagrams of cell displacement recorded one hour after setting up the Dunn chamber. Each point represents the position of a cell, which at time 0 is positioned at the intersection of the two axes. The × and Y axes are in μm. Labels: FIB - indicates the coverglass was coated with fibronectin, MCP-1 - monocyte chemoattractant protein-1, 300 - indicates 300 ng/ml concentration of MCP-1. Click here for file

Additional file 3 **Stimulation of TG WT and TG G93A microglia with 1 mM ATP**. (A) Intracellular calcium concentration ([Ca^2+^]_i_, μM) changes in NTG (WT) and TG WT microglia following extracellular stimulation with 1 mM ATP for 1 min. The period of ATP challenge is indicated with the bar. (B) Changes of [Ca^2+^]_i _in NTG (G93A) and TG G93A microglia following extracellular stimulation with 1 mM ATP for 1 min. The period of ATP challenge is indicated with the bar. (C) Area-under-the-curve (AUC) values (μM*s) for NTG (WT), TG WT, NTG (G93A) and TG G93A microglia. Data are mean ± SEM; Student's t test, *p = 0.0201 versus NTG (WT) microglia. (D) [Ca^2+^]_i _values (μM) recorded for NTG (WT), TG WT, NTG (G93A) and TG G93A microglia at 130 s of recording (10 s after initiation of 1 mM ATP challenge). Data are mean ± SEM; Student's t test, *p = 0.0381 versus NTG (WT) and **p = 0.0015 versus NTG (G93A) cells, n = three NTG (WT) mice (14 cells) and three TG WT mice (18 cells) from two litters, three NTG (G93A) mice (43 cells) and three TG G93A mice (38 cells) from two litters. Click here for file

## References

[B1] Brownell B Oppenheimer D Hughes J The central nervous system in motor neurone disease J Neurol Neurosurg Psychiatry 1970 33 338 357 10.1136/jnnp.33.3.338 5431724 PMC493478

[B2] Rowland L Diagnosis of amyotrophic lateral sclerosis J Neurol Sci 1998 160 Suppl 1 S6 24 9851643 10.1016/s0022-510x(98)00193-2

[B3] Shaw P Molecular and cellular pathways of neurodegeneration in motor neurone disease J Neurol Neurosurg Psychiatry 2005 76 1046 1057 10.1136/jnnp.2004.048652 16024877 PMC1739758

[B4] Bruijn L Miller T Cleveland D Unraveling the mechanisms involved in motor neuron degeneration in ALS Annu Rev Neurosci 2004 27 723 749 10.1146/annurev.neuro.27.070203.144244 15217349

[B5] Rosen D Siddique T Patterson D Figlewicz D Sapp P Hentati A Donaldson D Goto J O'Regan J Deng H Mutations in Cu/Zn superoxide dismutase gene are associated with familial amyotrophic lateral sclerosis Nature 1993 362 59 62 10.1038/362059a0 8446170

[B6] Andersen P Nilsson P Keränen M Forsgren L Hägglund J Karlsborg M Ronnevi L Gredal O Marklund S Phenotypic heterogeneity in motor neuron disease patients with CuZn-superoxide dismutase mutations in Scandinavia Brain 1997 120 Pt 10 1723 1737 9365366 10.1093/brain/120.10.1723

[B7] Haverkamp L Appel V Appel S Natural history of amyotrophic lateral sclerosis in a database population. Validation of a scoring system and a model for survival prediction Brain 1995 118 Pt 3 707 719 7600088 10.1093/brain/118.3.707

[B8] Bruijn L Becher M Lee M Anderson K Jenkins N Copeland N Sisodia S Rothstein J Borchelt D Price D Cleveland D ALS-linked SOD1 mutant G85R mediates damage to astrocytes and promotes rapidly progressive disease with SOD1-containing inclusions Neuron 1997 18 327 338 10.1016/S0896-6273(00)80272-X 9052802

[B9] Gurney M Pu H Chiu A Dal Canto M Polchow C Alexander D Caliendo J Hentati A Kwon Y Deng H Motor neuron degeneration in mice that express a human Cu, Zn superoxide dismutase mutation Science 1994 264 1772 1775 10.1126/science.8209258 8209258

[B10] Wong P Borchelt D Motor neuron disease caused by mutations in superoxide dismutase 1 Curr Opin Neurol 1995 8 294 301 10.1097/00019052-199508000-00008 7582045

[B11] Clement A Nguyen M Roberts E Garcia M Boillée S Rule M McMahon A Doucette W Siwek D Ferrante R Wild-type nonneuronal cells extend survival of SOD1 mutant motor neurons in ALS mice Science 2003 302 113 117 10.1126/science.1086071 14526083

[B12] Boillée S Yamanaka K Lobsiger C Copeland N Jenkins N Kassiotis G Kollias G Cleveland D Onset and progression in inherited ALS determined by motor neurons and microglia Science 2006 312 1389 1392 10.1126/science.1123511 16741123

[B13] Beers D Henkel J Xiao Q Zhao W Wang J Yen A Siklos L McKercher S Appel S Wild-type microglia extend survival in PU.1 knockout mice with familial amyotrophic lateral sclerosis Proc Natl Acad Sci USA 2006 103 16021 16026 10.1073/pnas.0607423103 17043238 PMC1613228

[B14] Almer G Teismann P Stevic Z Halaschek-Wiener J Deecke L Kostic V Przedborski S Increased levels of the pro-inflammatory prostaglandin PGE2 in CSF from ALS patients Neurology 2002 58 1277 1279 11971099 10.1212/wnl.58.8.1277

[B15] Henkel J Engelhardt J Siklós L Simpson E Kim S Pan T Goodman J Siddique T Beers D Appel S Presence of dendritic cells, MCP-1, and activated microglia/macrophages in amyotrophic lateral sclerosis spinal cord tissue Ann Neurol 2004 55 221 235 10.1002/ana.10805 14755726

[B16] Poloni M Facchetti D Mai R Micheli A Agnoletti L Francolini G Mora G Camana C Mazzini L Bachetti T Circulating levels of tumour necrosis factor-alpha and its soluble receptors are increased in the blood of patients with amyotrophic lateral sclerosis Neurosci Lett 2000 287 211 214 10.1016/S0304-3940(00)01177-0 10863032

[B17] Turner M Cagnin A Turkheimer F Miller C Shaw C Brooks D Leigh P Banati R Evidence of widespread cerebral microglial activation in amyotrophic lateral sclerosis: an [11C](R)-PK11195 positron emission tomography study Neurobiol Dis 2004 15 601 609 10.1016/j.nbd.2003.12.012 15056468

[B18] Troost D Claessen N van den Oord J Swaab D de Jong J Neuronophagia in the motor cortex in amyotrophic lateral sclerosis Neuropathol Appl Neurobiol 1993 19 390 397 10.1111/j.1365-2990.1993.tb00459.x 8278021

[B19] Kawamata T Akiyama H Yamada T McGeer P Immunologic reactions in amyotrophic lateral sclerosis brain and spinal cord tissue Am J Pathol 1992 140 691 707 1347673 PMC1886170

[B20] Sekizawa T Openshaw H Ohbo K Sugamura K Itoyama Y Niland J Cerebrospinal fluid interleukin 6 in amyotrophic lateral sclerosis: immunological parameter and comparison with inflammatory and non-inflammatory central nervous system diseases J Neurol Sci 1998 154 194 199 10.1016/S0022-510X(97)00228-1 9562310

[B21] Elliott J Cytokine upregulation in a murine model of familial amyotrophic lateral sclerosis Brain Res Mol Brain Res 2001 95 172 178 11687290 10.1016/s0169-328x(01)00242-x

[B22] Hemmer K Fransen L Vanderstichele H Vanmechelen E Heuschling P An in vitro model for the study of microglia-induced neurodegeneration: involvement of nitric oxide and tumor necrosis factor-alpha Neurochem Int 2001 38 557 565 10.1016/S0197-0186(00)00119-4 11290380

[B23] Hensley K Fedynyshyn J Ferrell S Floyd R Gordon B Grammas P Hamdheydari L Mhatre M Mou S Pye Q Message and protein-level elevation of tumor necrosis factor alpha (TNF alpha) and TNF alpha-modulating cytokines in spinal cords of the G93A-SOD1 mouse model for amyotrophic lateral sclerosis Neurobiol Dis 2003 14 74 80 10.1016/S0969-9961(03)00087-1 13678668

[B24] Weydt P Yuen E Ransom B Möller T Increased cytotoxic potential of microglia from ALS-transgenic mice Glia 2004 48 179 182 10.1002/glia.20062 15378658

[B25] Yoshihara T Ishigaki S Yamamoto M Liang Y Niwa J Takeuchi H Doyu M Sobue G Differential expression of inflammation- and apoptosis-related genes in spinal cords of a mutant SOD1 transgenic mouse model of familial amyotrophic lateral sclerosis J Neurochem 2002 80 158 167 10.1046/j.0022-3042.2001.00683.x 11796754

[B26] Sargsyan S Blackburn D Barber S Monk P Shaw P Mutant SOD1 G93A microglia have an inflammatory phenotype and elevated production of MCP-1 Neuroreport 2009 20 1450 1455 10.1097/WNR.0b013e328331e8fa 19752764 PMC2889291

[B27] Almer G Guégan C Teismann P Naini A Rosoklija G Hays A Chen C Przedborski S Increased expression of the pro-inflammatory enzyme cyclooxygenase-2 in amyotrophic lateral sclerosis Ann Neurol 2001 49 176 185 10.1002/1531-8249(20010201)49:2<176::AID-ANA37>3.0.CO;2-X 11220737

[B28] Drew P Xu J Storer P Chavis J Racke M Peroxisome proliferator-activated receptor agonist regulation of glial activation: relevance to CNS inflammatory disorders Neurochem Int 2006 49 183 189 10.1016/j.neuint.2006.04.003 16753239

[B29] Kriz J Nguyen M Julien J Minocycline slows disease progression in a mouse model of amyotrophic lateral sclerosis Neurobiol Dis 2002 10 268 278 10.1006/nbdi.2002.0487 12270689

[B30] Van Den Bosch L Tilkin P Lemmens G Robberecht W Minocycline delays disease onset and mortality in a transgenic model of ALS Neuroreport 2002 13 1067 1070 10.1097/00001756-200206120-00018 12060810

[B31] Zhu S Stavrovskaya I Drozda M Kim B Ona V Li M Sarang S Liu A Hartley D Wu D Minocycline inhibits cytochrome c release and delays progression of amyotrophic lateral sclerosis in mice Nature 2002 417 74 78 10.1038/417074a 11986668

[B32] Gurney M Cutting F Zhai P Andrus P Hall E Pathogenic mechanisms in familial amyotrophic lateral sclerosis due to mutation of Cu, Zn superoxide dismutase Pathol Biol (Paris) 1996 44 51 56 8734301

[B33] Ceballos-Picot I Nicole A Briand P Grimber G Delacourte A Defossez A Javoy-Agid F Lafon M Blouin J Sinet P Neuronal-specific expression of human copper-zinc superoxide dismutase gene in transgenic mice: animal model of gene dosage effects in Down's syndrome Brain Res 1991 552 198 214 10.1016/0006-8993(91)90084-9 1717112

[B34] Epstein C Avraham K Lovett M Smith S Elroy-Stein O Rotman G Bry C Groner Y Transgenic mice with increased Cu/Zn-superoxide dismutase activity: animal model of dosage effects in Down syndrome Proc Natl Acad Sci USA 1987 84 8044 8048 10.1073/pnas.84.22.8044 2960971 PMC299473

[B35] Avraham K Schickler M Sapoznikov D Yarom R Groner Y Down's syndrome: abnormal neuromuscular junction in tongue of transgenic mice with elevated levels of human Cu/Zn-superoxide dismutase Cell 1988 54 823 829 10.1016/S0092-8674(88)91153-1 2970304

[B36] Yarom R Sapoznikov D Havivi Y Avraham K Schickler M Groner Y Premature aging changes in neuromuscular junctions of transgenic mice with an extra human CuZnSOD gene: a model for tongue pathology in Down's syndrome J Neurol Sci 1988 88 41 53 10.1016/0022-510X(88)90204-3 2976083

[B37] Shibata N Asayama K Hirano A Kobayashi M Immunohistochemical study on superoxide dismutases in spinal cords from autopsied patients with amyotrophic lateral sclerosis Dev Neurosci 1996 18 492 498 10.1159/000111445 8940623

[B38] Deng H Shi Y Furukawa Y Zhai H Fu R Liu E Gorrie G Khan M Hung W Bigio E Conversion to the amyotrophic lateral sclerosis phenotype is associated with intermolecular linked insoluble aggregates of SOD1 in mitochondria Proc Natl Acad Sci USA 2006 103 7142 7147 10.1073/pnas.0602046103 16636275 PMC1447523

[B39] Ezzi S Urushitani M Julien J Wild-type superoxide dismutase acquires binding and toxic properties of ALS-linked mutant forms through oxidation J Neurochem 2007 102 170 178 10.1111/j.1471-4159.2007.04531.x 17394546

[B40] Hanisch U Kettenmann H Microglia: active sensor and versatile effector cells in the normal and pathologic brain Nat Neurosci 2007 10 1387 1394 10.1038/nn1997 17965659

[B41] Nimmerjahn A Kirchhoff F Helmchen F Resting microglial cells are highly dynamic surveillants of brain parenchyma in vivo Science 2005 308 1314 1318 10.1126/science.1110647 15831717

[B42] Davalos D Grutzendler J Yang G Kim J Zuo Y Jung S Littman D Dustin M Gan W ATP mediates rapid microglial response to local brain injury in vivo Nat Neurosci 2005 8 752 758 10.1038/nn1472 15895084

[B43] Raivich G Haas S Werner A Klein M Kloss C Kreutzberg G Regulation of MCSF receptors on microglia in the normal and injured mouse central nervous system: a quantitative immunofluorescence study using confocal laser microscopy J Comp Neurol 1998 395 342 358 10.1002/(SICI)1096-9861(19980808)395:3<342::AID-CNE6>3.0.CO;2-2 9596528

[B44] van Rossum D Hanisch U Microglia Metab Brain Dis 2004 19 393 411 15554430 10.1023/b:mebr.0000043984.73063.d8

[B45] Capani F Ellisman M Martone M Filamentous actin is concentrated in specific subpopulations of neuronal and glial structures in rat central nervous system Brain Res 2001 923 1 11 10.1016/S0006-8993(01)03189-4 11743966

[B46] Nolte C Möller T Walter T Kettenmann H Complement 5a controls motility of murine microglial cells in vitro via activation of an inhibitory G-protein and the rearrangement of the actin cytoskeleton Neuroscience 1996 73 1091 1107 10.1016/0306-4522(96)00106-6 8809827

[B47] Dibaj P Steffens H Zschuntzsch J Nadrigny F Schomburg ED Kirchhoff F Neusch C In Vivo Imaging Reveals Distinct Inflammatory Activity of CNS Microglia versus PNS Macrophages in a Mouse Model for ALS PLoS One 2011 6 e17910 10.1371/journal.pone.0017910 21437247 PMC3060882

[B48] Brakebusch C Fässler R beta 1 integrin function in vivo: adhesion, migration and more Cancer Metastasis Rev 2005 24 403 411 10.1007/s10555-005-5132-5 16258728

[B49] Breau M Pietri T Eder O Blanche M Brakebusch C Fässler R Thiery J Dufour S Lack of beta1 integrins in enteric neural crest cells leads to a Hirschsprung-like phenotype Development 2006 133 1725 1734 10.1242/dev.02346 16571628

[B50] Leone D Relvas J Campos L Hemmi S Brakebusch C Fässler R Ffrench-Constant C Suter U Regulation of neural progenitor proliferation and survival by beta1 integrins J Cell Sci 2005 118 2589 2599 10.1242/jcs.02396 15928047

[B51] Streit W Kreutzberg G Response of endogenous glial cells to motor neuron degeneration induced by toxic ricin J Comp Neurol 1988 268 248 263 10.1002/cne.902680209 3360987

[B52] Pearson H Payne B Cunningham T Microglial invasion and activation in response to naturally occurring neuronal degeneration in the ganglion cell layer of the postnatal cat retina Brain Res Dev Brain Res 1993 76 249 255 8149591 10.1016/0165-3806(93)90213-t

[B53] Thanos S The Relationship of Microglial Cells to Dying Neurons During Natural Neuronal Cell Death and Axotomy-induced Degeneration of the Rat Retina Eur J Neurosci 1991 3 1189 1207 10.1111/j.1460-9568.1991.tb00054.x 12106219

[B54] Hetz C Thielen P Matus S Nassif M Court F Kiffin R Martinez G Cuervo A Brown R Glimcher L XBP-1 deficiency in the nervous system protects against amyotrophic lateral sclerosis by increasing autophagy Genes Dev 2009 23 2294 2306 10.1101/gad.1830709 19762508 PMC2758741

[B55] Hsieh C Koike M Spusta S Niemi E Yenari M Nakamura M Seaman W A role for TREM2 ligands in the phagocytosis of apoptotic neuronal cells by microglia J Neurochem 2009 109 1144 1156 10.1111/j.1471-4159.2009.06042.x 19302484 PMC3087597

[B56] Takahashi K Prinz M Stagi M Chechneva O Neumann H TREM2-transduced myeloid precursors mediate nervous tissue debris clearance and facilitate recovery in an animal model of multiple sclerosis PLoS Med 2007 4 e124 10.1371/journal.pmed.0040124 17425404 PMC1851623

[B57] Ralevic V Burnstock G Receptors for purines and pyrimidines Pharmacol Rev 1998 50 413 492 9755289

[B58] Ohsawa K Irino Y Nakamura Y Akazawa C Inoue K Kohsaka S Involvement of P2X4 and P2Y12 receptors in ATP-induced microglial chemotaxis Glia 2007 55 604 616 10.1002/glia.20489 17299767

[B59] Inoue K The function of microglia through purinergic receptors: neuropathic pain and cytokine release Pharmacol Ther 2006 109 210 226 10.1016/j.pharmthera.2005.07.001 16169595

[B60] Tsuda M Tozaki-Saitoh H Inoue K Pain and purinergic signaling Brain Res Rev 2010 63 222 232 10.1016/j.brainresrev.2009.11.003 19931560

[B61] Lambert C Ase AR Seguela P Antel JP Distinct migratory and cytokine responses of human microglia and macrophages to ATP Brain Behav Immun 2010 24 1241 1248 10.1016/j.bbi.2010.02.010 20206681

[B62] Inoue K Purinergic systems in microglia Cell Mol Life Sci 2008 65 3074 3080 10.1007/s00018-008-8210-3 18563292 PMC11131657

[B63] McLarnon J Purinergic mediated changes in Ca2+ mobilization and functional responses in microglia: effects of low levels of ATP J Neurosci Res 2005 81 349 356 10.1002/jnr.20475 15948175

[B64] D'Ambrosi N Finocchi P Apolloni S Cozzolino M Ferri A Padovano V Pietrini G Carrì M Volonté C The proinflammatory action of microglial P2 receptors is enhanced in SOD1 models for amyotrophic lateral sclerosis J Immunol 2009 183 4648 4656 10.4049/jimmunol.0901212 19734218

[B65] Saura J Tusell J Serratosa J High-yield isolation of murine microglia by mild trypsinization Glia 2003 44 183 189 10.1002/glia.10274 14603460

[B66] Cashman N Durham H Blusztajn J Oda K Tabira T Shaw I Dahrouge S Antel J Neuroblastoma × spinal cord (NSC) hybrid cell lines resemble developing motor neurons Dev Dyn 1992 194 209 221 10.1002/aja.1001940306 1467557

[B67] Menzies F Cookson M Taylor R Turnbull D Chrzanowska-Lightowlers Z Dong L Figlewicz D Shaw P Mitochondrial dysfunction in a cell culture model of familial amyotrophic lateral sclerosis Brain 2002 125 1522 1533 10.1093/brain/awf167 12077002

[B68] Grosskreutz J Haastert K Dewil M Van Damme P Callewaert G Robberecht W Dengler R Van Den Bosch L Role of mitochondria in kainate-induced fast Ca2+ transients in cultured spinal motor neurons Cell Calcium 2007 42 59 69 10.1016/j.ceca.2006.11.010 17241659

[B69] Haastert K Grosskreutz J Jaeckel M Laderer C Bufler J Grothe C Claus P Rat embryonic motoneurons in long-term co-culture with Schwann cells--a system to investigate motoneuron diseases on a cellular level in vitro J Neurosci Methods 2005 142 275 284 10.1016/j.jneumeth.2004.09.003 15698667

[B70] Grynkiewicz G Poenie M Tsien R A new generation of Ca2+ indicators with greatly improved fluorescence properties J Biol Chem 1985 260 3440 3450 3838314

