# Supplementary figures and images for: A comparison of in vitro properties of resting SOD1 transgenic microglia reveals evidence of reduced neuroprotective function
Source: BMC Neurosci. 2011 Sep 23;12:91. doi: 10.1186/1471-2202-12-91 (PMC3191510; doi:10.1186/1471-2202-12-91)

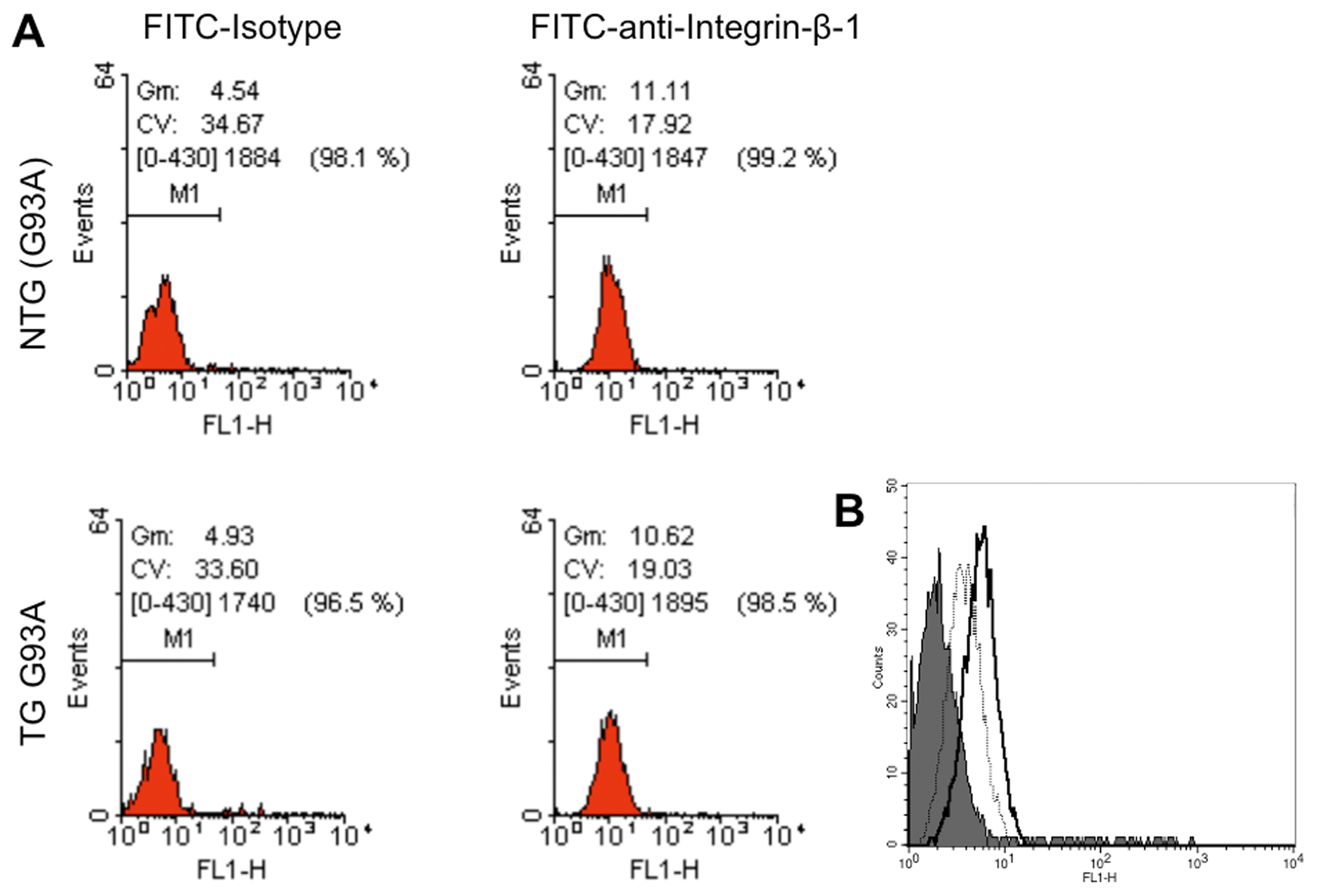

Supplement: Additional file 1 — Calculation of percent expression of integrin β-1 on primary microglia. (A) A representative example of flow cytometry histograms for NTG (G93A) and TG G93A microglia with FITC-isotype antibody or FITC-anti-integrin β-1 antibody is presented. Each histogram contains data on the geometric mean fluorescence (Gm), coefficient of variance (CV), marker position (0-430), number and percentage of cells under the marker. (B) An overlay of histograms from another independent experiment represents the FITC signal distribution for FITC-isotype antibody stained TG G93A microglia (filled curve), FITC-anti-integrin β-1 antibody stained TG G93A microglia (curve with dashed line) and FITC-anti-integrin β-1 antibody stained NTG (G93A) microglia (curve with solid line). The horizontal axis in (A) and (B), FL-1-H, is the detected signal intensity of FITC on a logarithmic scale. An example of percentage expression calculation is given below: 1. Under the marker M1 over 95% of the cells are detected, with the Gm for NTG (G93A) microglia of 11.11 with specific antibody, and 4.54 with isotype antibody. 2. For these NTG (G93A) microglia the integrin β-1 expression is 11.11 - 4.54 = 6.57, and for TG G93A cells the integrin β-1 expression is 10.62 - 4.93 = 5.69. These expression values are then converted to values on a linear scale. The values for NTG (G93A) are set as 100% expression. The values from TG (G93A) then converted to percent expression with respect to 100% of NTG (G93A) cells. [file 1471-2202-12-91-S1.PNG]

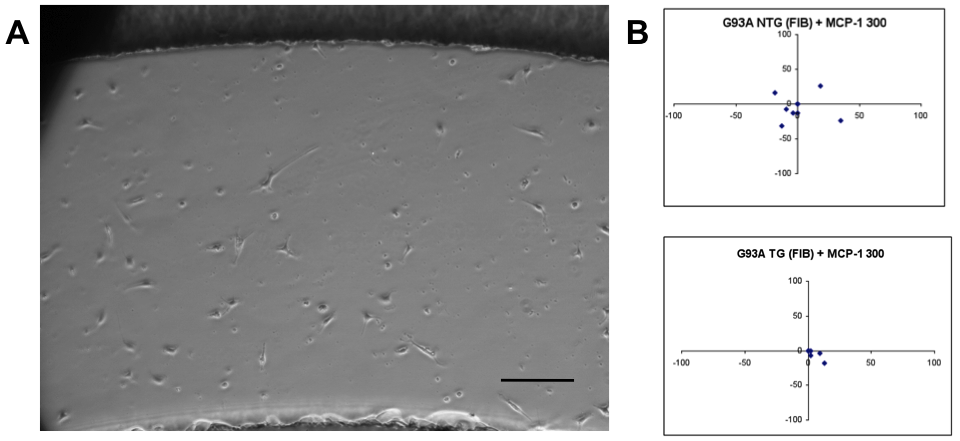

Supplement: Additional file 2 — Migration of TG WT and TG G93A microglia towards the chemoattractant MCP-1: (A) A representative image of microglial cells on fibronectin-coated coverglass overlaying a Dunn migration chamber with cells facing the chamber. The image was taken on the LEICA Microsystems AF6000LX microscope on a x10 objective, with environmental control to maintain the slide at 37°C. To record migration, images were taken every 3 minutes for 1 hour using Leica software. At the top of the picture is the edge of the outer well, which was filled with medium containing MCP-1. The lower edge is the inner well containing normal medium. Scale bar = 50 μm. (B) An example of analysis of migrated distance represents vector diagrams of cell displacement recorded one hour after setting up the Dunn chamber. Each point represents the position of a cell, which at time 0 is positioned at the intersection of the two axes. The × and Y axes are in μm. Labels: FIB - indicates the coverglass was coated with fibronectin, MCP-1 - monocyte chemoattractant protein-1, 300 - indicates 300 ng/ml concentration of MCP-1. [file 1471-2202-12-91-S2.PNG]

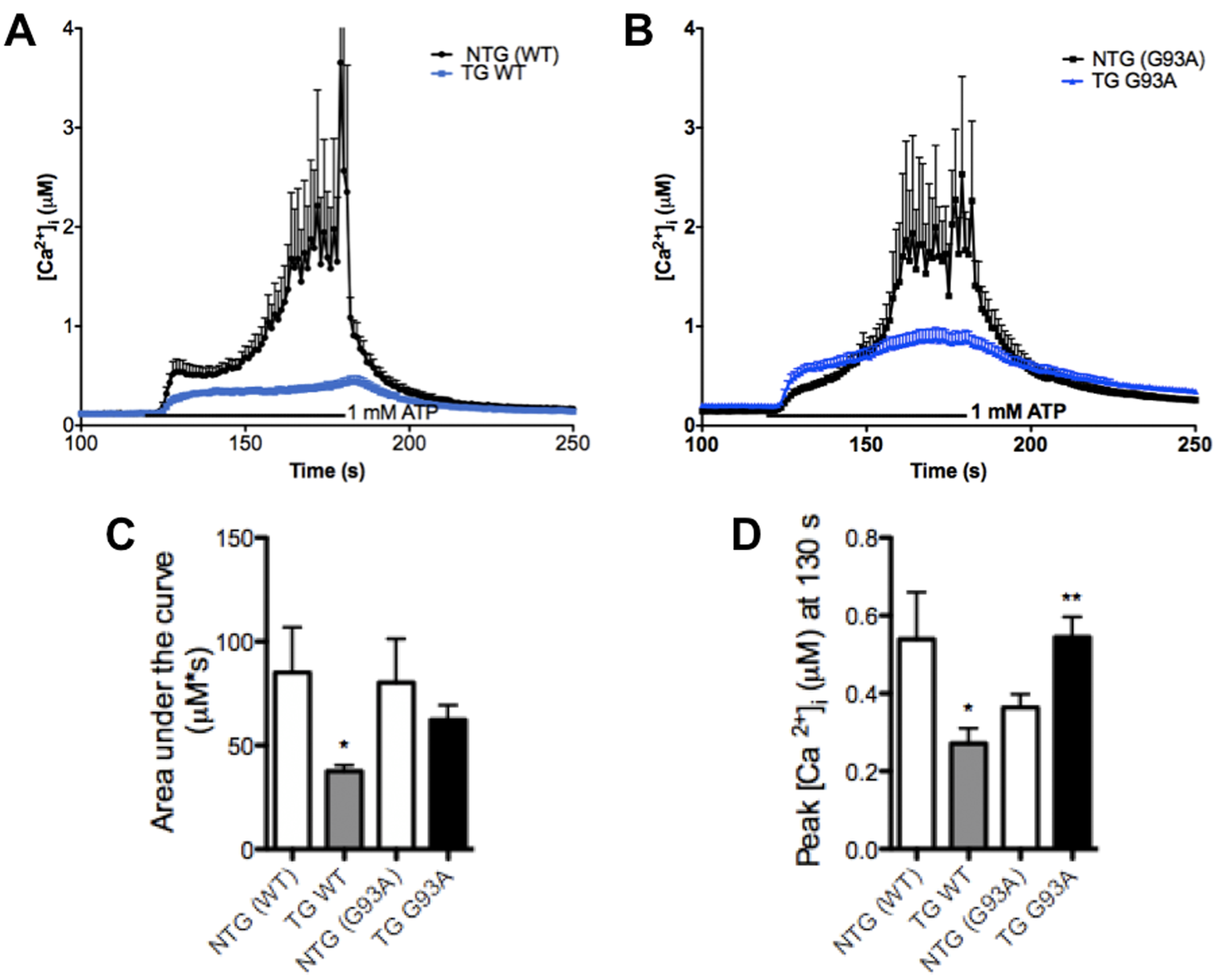

Supplement: Additional file 3 — Stimulation of TG WT and TG G93A microglia with 1 mM ATP. (A) Intracellular calcium concentration ([Ca2+]i, μM) changes in NTG (WT) and TG WT microglia following extracellular stimulation with 1 mM ATP for 1 min. The period of ATP challenge is indicated with the bar. (B) Changes of [Ca2+]i in NTG (G93A) and TG G93A microglia following extracellular stimulation with 1 mM ATP for 1 min. The period of ATP challenge is indicated with the bar. (C) Area-under-the-curve (AUC) values (μM*s) for NTG (WT), TG WT, NTG (G93A) and TG G93A microglia. Data are mean ± SEM; Student's t test, *p = 0.0201 versus NTG (WT) microglia. (D) [Ca2+]i values (μM) recorded for NTG (WT), TG WT, NTG (G93A) and TG G93A microglia at 130 s of recording (10 s after initiation of 1 mM ATP challenge). Data are mean ± SEM; Student's t test, *p = 0.0381 versus NTG (WT) and **p = 0.0015 versus NTG (G93A) cells, n = three NTG (WT) mice (14 cells) and three TG WT mice (18 cells) from two litters, three NTG (G93A) mice (43 cells) and three TG G93A mice (38 cells) from two litters. [file 1471-2202-12-91-S3.PNG]
